# Geospatial analysis of cholera outbreak in Lusaka, Zambia, between 2023 and 2024

**DOI:** 10.1186/s41182-025-00718-4

**Published:** 2025-03-28

**Authors:** William Ngosa, Tadatsugu Imamura, Nyuma Mbewe, Joseph Seriki, Oscar Nzila, Fred Mfune, Godfrey Zulu, Chomba Mulando, Tizha Chiluba, Luo Miyanda, Agness Phiri, Lucy Sichone, Galion Mwape, Kapambwe Mulenga, Charles Chileshe, Nawa Mabuku, Dabwiso Banda, Fangyu Yan, Taro Kamigaki, Roma Chilengi, Nyambe Sinyange

**Affiliations:** 1 Zambia National Public Health Institute, 10101 Lusaka, Zambia; 2 Lusaka Provincial Health Office, 10101 Lusaka, Zambia; 3 Japan International Cooperation Agency, Tokyo, 102-8012 Japan; 4 Center for Postgraduate Education and Training, National Center for Child Health and Development, Tokyo, 157-8535 Japan; 5 International Federation of Red Cross and Red Crescent Societies, 10101 Lusaka, Zambia; 6 Levy Mwanawasa Medical University, 10101 Lusaka, Zambia; 7 Center for Surveillance, Immunization, and Epidemiologic Research, National Institute of Infectious Diseases, Tokyo, 162-8640 Japan; 8 Project for Strengthening Laboratory-Based Surveillance for Infectious Diseases, Japan International Cooperation Agency, Stand 1186, Corner of Chaholi and Addis Ababa Roads, 10101 Lusaka, Zambia

**Keywords:** Geospatial, *Vibrio cholerae*, Outbreak response, Geographic information system, Oral rehydration points

## Abstract

**Background:**

Cholera outbreaks have plagued Zambia for decades, with Lusaka district, the capital, being particularly vulnerable. Although the lack of sanitary toilet facilities and inadequate drainage systems were shown to be associated with the high cholera incidence in the early 2000s, it is unknown whether these environmental risk factors persisted in the outbreak that occurred in 2023–2024, which turned out to be the largest outbreak in the country’s history. We investigated the geospatial patterns of cholera cases and associated environmental factors during the October 2023 to March 2024 cholera outbreak.

**Methods:**

We conducted a geospatial analysis of the suspected cholera cases in Lusaka district, comprising seven constituencies and 94 townships. Patient information and geocoordinates were collected from suspected cases using electronic surveillance tools. The space–time scan statistics was performed to detect spatial and temporal clusters of cases. Spearman's rank correlation coefficient, were employed to examine the relationship between cholera incidence and various environmental factors, including access to Water, Sanitation, and Hygiene (WASH) facilities and equipment.

**Results:**

Over the study period, 4,591 suspected cholera cases with geocoordinate data were identified, with incidence rates varying across the constituencies. Median cholera incidence (IQR) was 0.55 (0.27–1.44) in Lusaka, with higher incidence rates observed in unplanned residential areas. After the first case identification in Kanyama, cases and clusters were observed in different parts of Lusaka. Among 94 townships in Lusaka, cholera-suspected cases were identified in 86 of them. Among environmental factors analyzed for associations with the high cholera incidence, the proportion of individuals without soap and detergent at home (ρ = 0.457, *p* < 0.001) and those without water for hand washing at home (ρ = 0.421, *p* < 0.001) were significantly associated with increased cholera incidence.

**Conclusion:**

The findings underscore the significance of environmental factors in cholera transmission, particularly in unplanned residential areas with inadequate access to WASH facilities which persist in the area. Improving WASH infrastructure and implementing tailored public health strategies, particularly for high-risk areas (e.g., unplanned residential areas), are crucial for mitigating cholera outbreaks in Lusaka District.

**Supplementary Information:**

The online version contains supplementary material available at 10.1186/s41182-025-00718-4.

## Background

Cholera is an acute gastroenteritis caused by a bacterial agent *Vibrio cholerae* (*V. cholerae*) [1]. The bacteria still remain a pathogen of public health concern in Africa, and a total of 335,059 cholera cases, including 6197 deaths, were reported in the World Health Organization Regional Office for Africa between January 1, 2022, and March 3, 2024 [2]. Zambia has been affected by repeated epidemics of cholera since 1977, and over 30 cholera outbreaks with over 10,000 cases cumulatively were reported in the country between 1977 and 2019 [3]. The major cholera outbreak which occurred in the capital Lusaka between 2017 and 2018 caused more than 5,000 cases and 90 deaths [4]. During the outbreak in 2017–2018, majority of cases and deaths were reported from low-income residential areas, including Kanyama subdistrict which had been the origin of the recent cholera outbreaks in Lusaka in 2006, 2016, and 2017–2018 [5–7]. Such geographical distribution of cases was assumed to be linked to environmental factors, including the vulnerable water, sanitation, and hygiene (WASH) system in those areas [6, 8]. Findings from our previous study shows that pit latrines and toilet outside houses were significantly correlated with the increased number of cases in Kanyama subdistrict during the outbreak in 2017–18 [7]. It highlighted the importance of investigating environmental factors of infectious disease epidemics to identify high priority areas for public health interventions.

On October 15, 2023, the primary cases of the cholera outbreak in Lusaka Province were reported from Kanyama subdistrict [9]. The number of cases increased rapidly across the province, and it reached its peak on January 8, 2024 [10, 11]. As of April 3, 2024, a total of 22,565 cases and 725 deaths had been reported nationwide since the beginning of the outbreak, and it became the largest cholera epidemic in Zambia since 1977 [3, 12]. Among these cases, 14,492 cases and 514 deaths were recorded in Lusaka District, the epicenter of the current outbreak [10]. Despite the prompt need to identify high-risk areas and factors associated with cholera transmission in the current outbreak, geographical distribution patterns of cholera cases and environmental factors associated with such patterns in Lusaka District is still not fully understood. Sasaki et al. have previously demonstrated that the lack of latrines and drainage systems had a significant association with the high incidence of cholera during the outbreaks in Lusaka in the early 2000s [6, 8]. However, it is of public health significance to evaluate if such WASH related risk factors still persistent in the current outbreak, after the city development and intensive interventions to improve water supply, sanitation facilities, and drainage systems that occurred in Lusaka in the past decades [13–15].

In this study, we aimed to assess the geographical distribution patterns and associated environmental factors during the cholera outbreak in Lusaka District between October, 2023 and March, 2024.

## Methods

### Study site

**Fig. 1:**
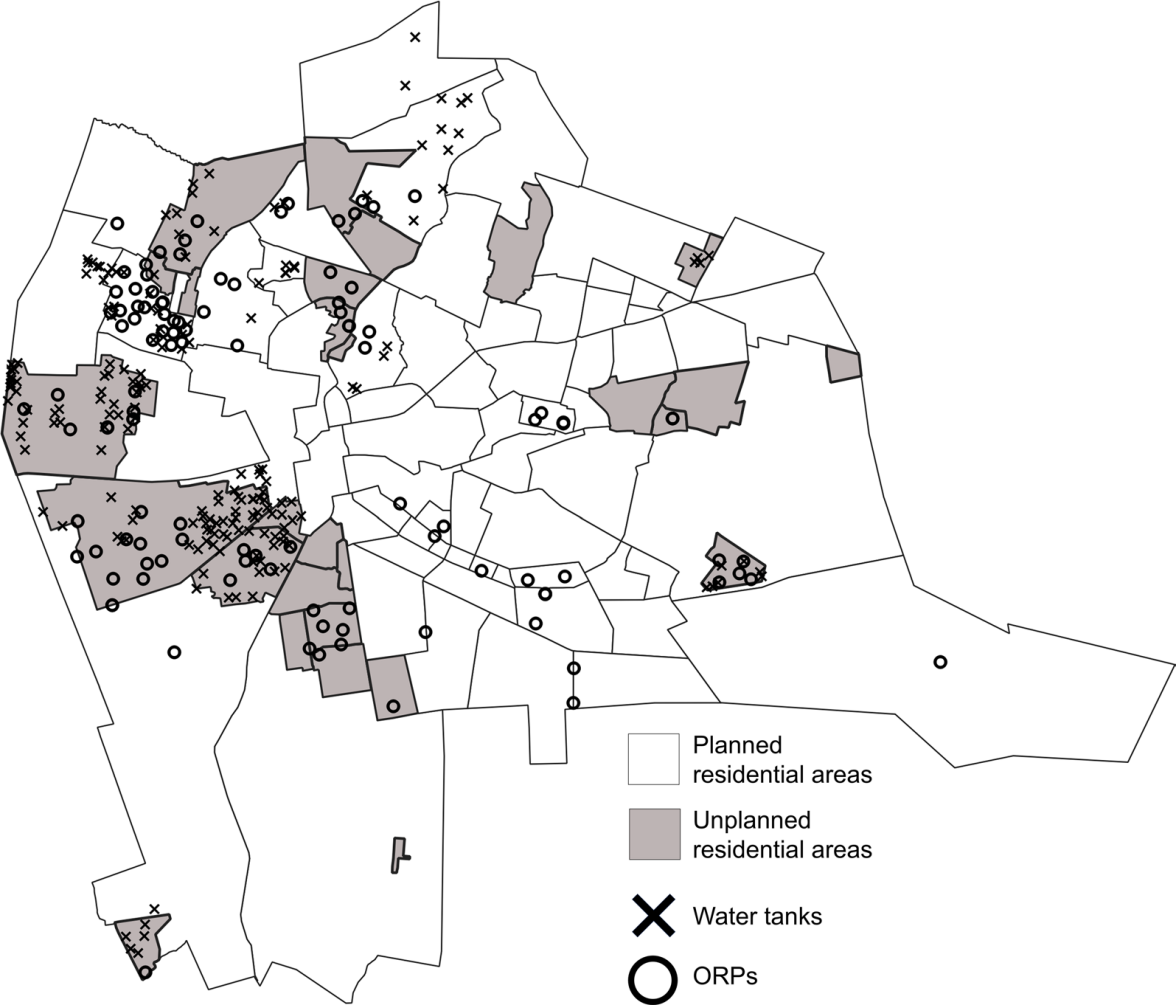
Geographical distribution of the townships, planned/unplanned residential areas, water tanks, and oral rehydration points in Lusaka, Zambia, October 2023- March 2024. Locations of the townships, planned/unplanned residential areas, water tanks, and oral rehydration points (ORPs) were indicated

The study was conducted in Lusaka District; the capital of Zambia. Lusaka District has a population of over 2,200,000 [16]. The district consists of 7 constituencies including Lusaka Central, Chawama, Kabwata, Kanyama, Mandevu, Matero, and Munali [17]. Each constituency is further divided into multiple lower administrative divisions, called a township. In total, there are 94 townships in Lusaka District, including planned and unplanned residential areas (Fig. 1) [18]. Planned residential areas were officially created by the Government Planning Authority, unlike unplanned residential areas (Fig. 1) [19]. Unplanned residential areas are characterized by a high population density and poor access to safe water supplies [19, 20]. In this study, the administrative unit of township was used for the spatial analysis.

There are four seasons which include hot and dry season (mid-August to mid-November), wet rainy season (mid-November to April), a cool dry season (May to mid-August), and rainy seasons. The rainy season starts in November and ends in April, which causes severe flooding, especially in unplanned residential areas in Lusaka [17, 21].

### Patient data collection

During the cholera outbreak in Lusaka, the surveillance officers of the Ministry of Health, Zambia (MOH) and Zambia National Public Health Institute (ZNPHI) conducted case investigations on individuals who suspected of cholera infection [22]. Based on the National Cholera Surveillance Guideline, cholera-suspected cases were defined as individuals who presented more than 3 times of watery stools within 24 h [22]. During the case investigations of MOH and ZNPHI for suspected cases, patient information and geocoordinate data at houses of cholera cases were collected by physical visits to these houses after informed consent was given by the patients or their guardians. Geocoordinate data were captured using the electronic Integrated Disease Surveillance and Response (eIDSR) as part of routine surveillance [23]. We conducted a retrospective data analysis on the patient information and geocoordinate data collected from suspected cases between October 14, 2023, and March 20, 2024.

### Water point data collection

Water tanks and the oral rehydration points (ORPs) were established in the communities of Lusaka District by a multisectoral team led by the Disaster Management and Mitigation Unit (DMMU). Water tank installations commenced from December 3, 2023, to March 20 2024, and ORP deployment kicked off from December 15, 2023. Water tanks and ORPs installations started from areas which was reporting a large number of cholera cases and low-income residential areas (e.g., compounds) where the water supply and sanitation systems were insufficient [10]. The geocoordinate data of the location of water tanks and ORPs were collected by ZNPHI using a koboToolbox designed tool, with the aim of monitoring their deployment in the community, in association with the spreading pattern of the outbreak in Lusaka.

### Environmental data collection

 Township boundaries and geospatial distribution of associated environmental factors were obtained from an open access data source of the Zambia Data Hub as previously described [24, 25]. We included a total of 5 WASH-related factors that was available in the Zambia Data Hub to explore associations between the cholera incidence and the geospatial distributions of access to safe water, hand hygiene facilities/equipment, and sanitary toilet facilities. Other factors, including the population density and the literacy of residents, were included, because they were unique characteristics of the unplanned residential areas, which were suspected for associations with the increased cholera cases in Lusaka [19, 20]. Associated factors and their definitions are listed in Supplementary Table 1.

### Geospatial maps

We generated digital maps showing the geospatial distribution of cholera cases and associated spatial factors by epidemiological weeks (EW) using QGIS version 3.10 A Coruña. Counting of the number of cholera cases, water tanks, and ORPs per township, integration of raster and township polygon data, and calculation of incidence (i.e., number of cholera cases per 1000 population in each township), area and population sizes of each township within Lusaka District boundary was conducted with QGIS. A Microsoft Excel sheet containing the cholera incidence, population density (/10^3^*km^2^), the number of water tanks and ORPs per area size (/10^7^*km^2^), and the proportion of individuals with the associated factors (%) of the 94 townships in Lusaka was generated and used for analysis.

For detection of spatial and temporal clusters of cholera cases, the space–time scan statistics was performed using SaTScan^™^ v10.1.3, to analyze geocoordinate data of houses of cholera cases within the Lusaka District boundary [26]. Discrete Poisson regression was selected as the probability model. The maximum spatial window area was set at 30% of the population at risk by referring to Gini coefficient among other window sizes [27]. Maximum Monte Carlo permutation was 999 in reference to previous studies [28, 29]. Generated maps showing the detected clusters were overlaid on the map showing the geographical distribution of cases (cholera incidence per township) using QGIS.

### Statistical analysis

Statistical analysis was conducted using R ver.4.3.2 (R Foundation for Statistical Computing, Vienna, Austria). The Spearman´s rank correlation coefficient was performed to calculate the correlation (the Spearman’s rho; ρ) between the cholera incidence and associated factors in continuous variables. Correlations between the number of water tanks and ORPs and the cholera incidence were evaluated by calculating the coefficient between the number of water tanks and ORPs per km^2^ (* 10^7^) and the cholera incidence per 1,000 in each township between December 15, 2023 and March 20, 2024. The Spearman’s ρ being equal to or greater than 0.4 was regarded as a strong correlation, ρ being equal to or greater than 0.3 was regarded as a moderate correlation, and ρ being equal to or greater than 0.2 was regarded as a weak correlation [30]. A p-value less than 0.05 was considered as statistically significant in all analyses.

### Ethical approval

The secondary use of the patient data, which was collected as part of the public health response of MOH and ZNPHI, for analysis and publication was approved by the National Health Research Authority (reference number NHRA-1565/18/09/2024). For study purposes, no individually identifiable data were included in the analysis.

## Results

### Study population

**Fig. 2:**
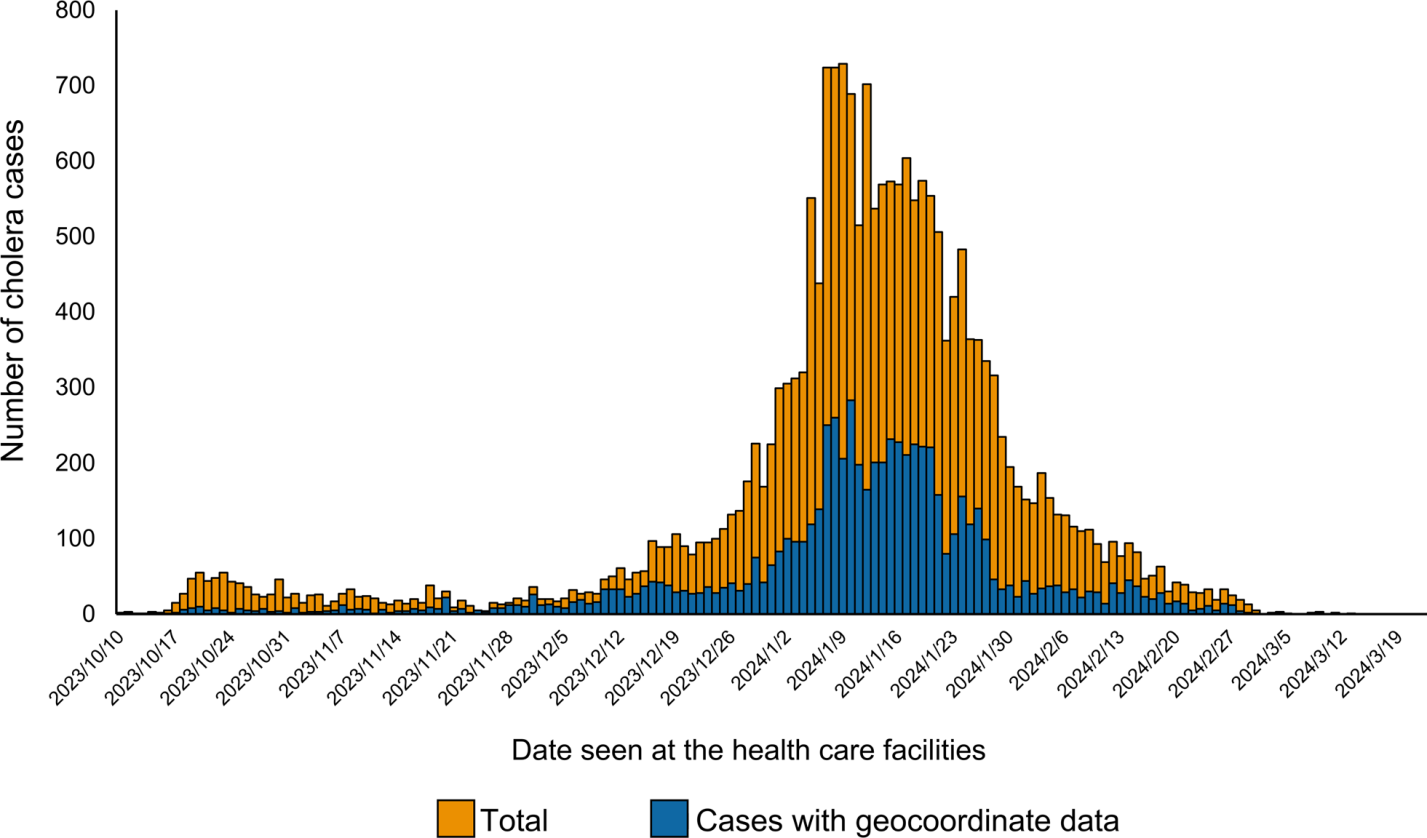
Temporal distribution of cholera cases in Lusaka District, October 2023–March 2024. Bar graphs show the number of cholera cases in total (orange) and those with geocoordinate data (blue). Line graph indicates the proportion of cases with geocoordinate data (red line)

A total of 4,591 cholera-suspected cases with geocoordinate data of their houses were identified between October 14, 2023, and March 20, 2024. This represented 28.4% (4,591/16,146) of the total cholera-suspected cases reported from Lusaka District in the same period (Supplementary Table 2). Number of cases rapidly increased from mid-December, reached its peak in early-January, and gradually decreased towards late-February, which showed a similar pattern with the epidemic curve of the total cases (Fig. 2). Among the 4591 cases with geocoordinates, males represented with 2554 (55.6%), while 2037 (44.3%) were female. Median (interquartile range; IQR) age of the patients was 23.0 (8.0–33.0). One hundred and nineteen cases (2.6%, 119/4,591) were fatal.

### Geospatial distribution of cholera-suspected cases

**Fig. 3:**
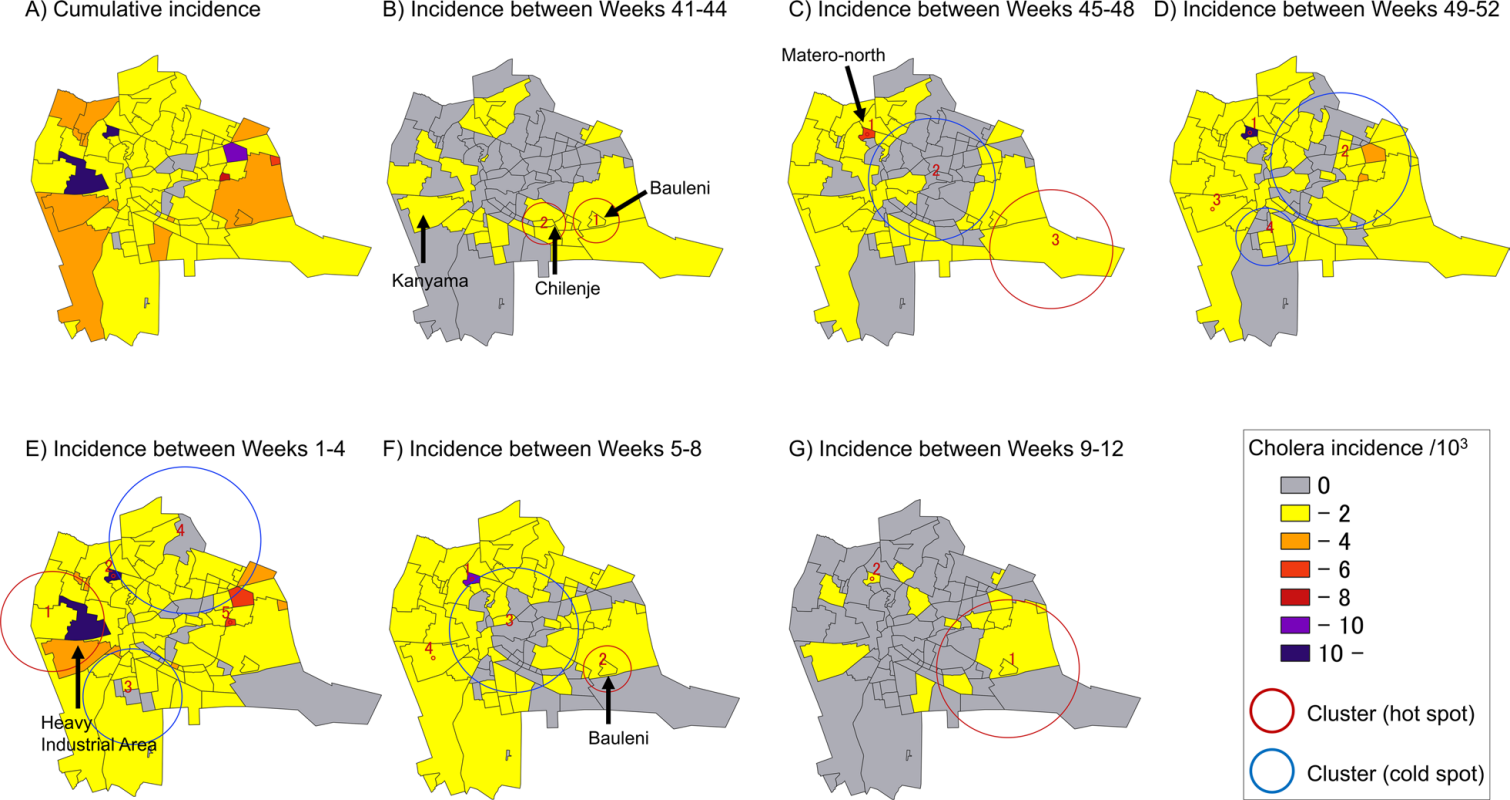
Cholera incidence and geographical distribution of clusters by epidemiological weeks in Lusaka District, October 2023–March 2024. The cumulative incidence of cholera-suspected cases (panel **A**) and the incidence and space–time clusters of cholera-suspected cases per township during the 4-epidemiological week period (panels **B**–**G**) is indicated

Among 94 townships in Lusaka, cholera-suspected cases (*n* = 4,201) were identified in 86 of them (91.5%, 86/94) (Fig. 3A). A total of 390 cases were first identified in health care facilities in Lusaka District, however, their houses were located outside the district boundary, therefore, excluded from analysis. Among these 86 townships, 26 (30.2%) were unplanned residential areas, where 2484 cholera-suspected cases (59.1%, 2484/4201) were identified. Median cholera incidence per 1000 (IQR) was 0.52 (0.27–1.44) in Lusaka District. The incidence per 1000 (median, IQR) was significantly larger in unplanned residential areas (0.86, 0.17–2.35) than in planned residential areas (0.47, 0.29–0.96) (*p* < 0.001).

Between EW41-44, the crude number of less than 20 cases per township was reported from 28 townships in Lusaka District, among which 12 were unplanned residential areas, including Kanyama where the primary cases were identified (Fig. 3B). The incidence per 1000 was highest in Chilenje (0.32/10^3^), followed by Bauleni (0.26/10^3^) (Fig. 3B). Number of cases increased across Lusaka District, particularly in Kanyama and Matero-north; major compounds in the district, between EW45-52 (Fig. 3C, D). Number of cases overshot between EW1-4, and 14 townships, including 9 unplanned residential areas, reported the crude number of over 50 cases per township (Fig. 3E). In EW1-4, the incidence per 1,000 was highest in Matero-north (16.7/10^3^) followed by Heavy Industrial Area (13.4/10^3^) (Fig. 3E). Between EW5-8, number of new cases reduced compared to EW1-4, although 3 townships, including Kanyama, Matero-north, and Bauleni, continued reporting the crude number of over 50 cases per township (Fig. 3F). Number of townships reporting new cases reduced to 16, with less than the crude number of 20 cases per township, between EW 9–12 (Fig. 3G).

### The space–time cluster of cholera in Lusaka between EW41, 2023, and EW12, 2024

Between EW 41–44, 2 statistically significant space–time clusters were identified in Lusaka District (Fig. 3B, Table 1). The first cluster was centered by Bauleni from October 29 to November 4 and there were 11 cases observed with a relative risk (RR) 34.2 compared to other areas. The second cluster was centered by Chilenje, October 22–28, with other 6 townships. There were 10 cases observed and RR was 15.5 compared to other areas. Between EW45-48, 2 clusters were observed, including the one centered by Matero North, November 19–25, with 27 cases observed and RR 234 (Fig. 3C, Table 1). The second cluster, November 5–11, included New Kasama and Bauleni, where 10 cases were observed and RR was 10.1.

**Table 1:** Space–time scan statistics of cholera cases in Lusaka District, 2023–2024

Cluster	Townships	Number of townships	Duration	*p*	LLR*	Radius (km)	Observed	Expected	O/E**	RR***
Epidemiologic week 41–44 (2023)										
1	Bauleni	2	Oct 29–Nov 4	< 0.001	27.309486	2.1	11	0.38	29.2	34.2
2	Chilenje	7	Oct 22–Oct 28	< 0.001	17.375077	1.98	10	0.74	13.48	15.46
Epidemiologic week 45–48 (2023)										
1	Matero North	1	Nov 19–Nov 25	< 0.001	118.310812	0	27	0.13	200.59	234.48
2	Mass Media	48	Nov 19–Nov 25	< 0.001	14.240036	5.79	0	13.71	0	0
3	New Kasama	2	Nov 5–Nov 11	< 0.001	13.910586	5.63	10	1.04	9.63	10.12
Epidemiologic week 49–52 (2023)										
1	Matero North	1	Dec 24–Dec 30	< 0.001	169.293172	0	48	0.54	88.55	94.55
2	Golf Course	41	Dec 3–Dec 9	< 0.001	39.615821	6.35	3	49.51	0.061	0.057
3	Kanyama	1	Dec 24–Dec 30	< 0.001	33.768203	0	66	20.22	3.26	3.48
4	Chawama	10	Dec 24–Dec 30	< 0.001	20.894636	2.7	2	27.7	0.072	0.07
Epidemiologic week 1–4 (2024)										
1	Garden Park	8	Jan 15–Jan 21	< 0.001	204.9501	4.55	406	129.15	3.14	3.56
2	Matero North	1	Jan 15–Jan 21	< 0.001	133.412344	0	54	1.75	30.81	31.47
3	John Howard	17	Jan 22–Jan 28	< 0.001	87.703035	4.34	9	117.4	0.077	0.073
4	Foxdale	23	Jan 15–Jan 21	< 0.001	85.747024	6.66	23	148.35	0.16	0.15
5	Kalikiliki	1	Jan 8–Jan 14	< 0.001	35.959176	0	30	3.79	7.91	7.99
Epidemiologic week 5–8 (2024)										
1	Matero North	1	Feb 5–Feb 11	< 0.001	96.094022	0	30	0.47	64.37	67.47
2	Bauleni	2	Feb 12–Feb 18	< 0.001	63.90688	2.1	40	3.32	12.04	12.77
3	Rhodes Park	45	Feb 19–Feb 25	< 0.001	49.345321	5.67	0	47.5	0	0
4	Kanyama	1	Jan 29–Feb 4	< 0.001	48.650669	0	71	17.38	4.08	4.47
Epidemiologic week 9–12 (2024)										
1	Bauleni	18	Feb 26–Mar 4	< 0.001	31.215855	6.25	23	1.83	12.55	27.32
2	Matero North	1	Feb 26–Mar 4	< 0.001	22.210034	0	6	0.033	183.26	214.5

^*^ LLR; log likelihood ratio

^**^ O/E; observed per expected

^***^ RR; relative risk

Between EW49-52, 2 clusters were observed in Matero North and Kanyama from December 24 to 30 (Fig. 3D, Table 1). A total of 48 and 66 cases were observed and RR was 94.6 and 3.48, respectively.

Between EW1-4, a total of 3 clusters were identified (Fig. 3E, Table 1). The largest cluster centered by Golden Park, January 15–21, was connected with other 7 townships including Heavy Industrial Area, Kanyama, Lusaka West, George, Paradise, Lilanda, and Desai (Fig. 3E, Table 1). The second cluster, January 15–21, was centered by Matero North (observed 54, RR 31.5) and the third cluster, January 8–14, was centered by Kalikiki (observed 30, RR7.99).

Between EW5-8, Matero North was identified as a cluster from February 5 to 11, with 30 observed cases and RR 67.5, which was followed by the cluster centered by Bauleni (observed 40, RR 12.8), February 12–18, and Kanyama (observed 71 cases, RR 4.47), January 29—February 4 (Fig. 3F, Table 1).

Between EW9-12, cases were accumulated in the cluster centered by Bauleni (observed 6.25, RR 27.3) from February 26 to March 4. Matero North remained to be identified as a center of the cluster between the same period, with 6 observed cases and RR 214 (Fig. 3G, Table 1).

### Environmental factors associated with cholera incidence

A total of 8 environmental factors were explored for correlations with the cholera incidence per 1000 (Table 2). Among them, the proportion of individuals without soap and detergent at home (ρ = 0.457, *p* < 0.05) and those without water for hand washing at home (ρ = 0.421, *p* < 0.05) was strongly correlated with increased cholera incidence per 1000 (Table 2). Proportion of females with literacy showed a negative and strong correlation with cholera incidence per 1000 (ρ = − 0.418, *p* < 0.05). The proportion of individuals who share toilets with others showed an weak correlation with the cholera incidence per 1000 (ρ = − 0.230, *p* = 0.03). Other 4 environmental factors were not significantly correlated with the cholera incidence per 1000 (Table 2).

**Table 2:** Environmental factors (Demographic Health Survey 2018) associated with cholera incidence in Lusaka, Zambia, October 2023–March 2024

Factors	Spearman’s rank correlation coefficient	*p*-value
Population density [/10^3^ km^2^]	0.077	0.46
Proportion of females with literacy	− 0.418	< 0.05
Proportion of males with literacy	− 0.018	0.86
Proportion of individuals without soap/detergent at home	0.457	< 0.05
Proportion of individuals without piped-in drinking water at home	0.167	0.11
Proportion of individuals without water for hand washing at home	0.421	< 0.05
Proportion of individuals who require more than 30 min to obtain water	0.178	0.09
Proportion of individuals who share toilets with others or do not have toilets at home	0.230	0.03

### Geographical distribution of water tanks and ORPs

During the outbreak, a total of 212 water tanks and 117 ORPs were established in Lusaka District (Fig. 1). Among the 94 townships, water tanks and ORPs were established in 20 (21.3%) and 32 (34.0%) of them, respectively. The median (IQR) number of water tanks per area was 8.2 (5.1–22.4) and that of ORPs was 0.0 (0.0–5.6). Among the 20 townships where water tanks were established, the number of water tanks per area showed a positive correlation with the cholera incidence per 1,000 (ρ = 0.384, *p* < 0.05). The number of ORPs per area did not show a positive correlation with the cholera incidence per 1,000 (ρ = 0.128, *p* = 0.22).

## Discussion

We reported the geographical distribution patterns of cholera cases and associated environmental factors in Lusaka District during the outbreak between October 15, 2023, and March 22, 2024.

Our results showed that cholera incidence was significantly higher in unplanned residential areas than planned residential areas in the current outbreak. Notably, the current outbreak followed a similar spreading pattern as the previous outbreak in 2017-18, in which majority of cases were reported from low-income residential areas (e.g., compounds) [4]. This suggests that similar areas of Lusaka District, particularly low-income residential areas including unplanned residential areas, have still remained vulnerable against cholera transmission. Such geographical distribution pattern was assumed to be because environmental risk factors, such as inadequate access to WASH related facilities/items, particularly those needed for hand hygiene practices (i.e., soap, detergent, and water for hand washing at home), persisted in those areas. Our findings were in line with previous studies from different parts of the world, which have shown the importance of improved water and sanitation systems in reduction of the cholera incidence [31–33]. This highlighted the importance of continuous efforts to improve and maintain the water and sanitation systems in those high-risk areas.

During the outbreak, the distribution of water tanks and ORPs were positively correlated with the cholera incidence. This was assumed to be the reflection of the fact that water tanks and ORPs were strategically established in areas reporting a large number of cases and those which were in need of immediate public health interventions to suppress the transmission. During the outbreak, the WASH team strategically selected areas and numbers of water tanks and ORPs to be mounted based on the maps showing the geographical distribution of cholera cases, which were generated and updated daily by the geospatial analysis team [34]. Such multisectoral approach in the cholera incident management system (IMS) was assumed to have contributed to enable timely case-area target intervention (CATI) for such high-risk areas [35].

Notably, Kanyama subdistrict was again the origin of the cholera outbreak in 2023-24, as were the previous outbreaks in 2006, 2016, and 2017-18 [5–7]. The underlying mechanism of how cholera was introduced into Kanyama subdistrict during the current outbreak was not clarified in this study. However, it is assumed that cholera transmission spread rapidly in Kanyama subdistrict after the introduction, due to its vulnerable water and sanitation systems as we previously reported [7]. In addition to the effort to improve WASH in those high-risk areas, capacities to detect early clusters of cholera cases is equally important [35, 36]. Based on the public health significance of Kanyama subdistrict for cholera emergence in Lusaka, ZNPHI launched a new surveillance program to monitor epidemiology and etiology of diarrheal disease cases in multiple sentinel surveillance sites across the country, among which Kanyama constituency site was launched as one of the high priority sites in December, 2023 [37] Details of the new diarrheal disease surveillance will be described elsewhere.

Although the primary cases of the outbreak were identified from Kanyama, the western side peri-urban area of Lusaka District, the space–time scan analysis has identified clusters of cholera cases also in the east side of the district (e.g., Bauleni and Chilenge) in the early phase of the outbreak. Additional clusters were identified in the west of the Lusaka District as the outbreak evolved. It highlights the importance of preventing spill-over of cholera from the origin (e.g., Kanyama) to other areas in the district, and monitoring for the emergence of suspected cases not only in epicenter.

In this study, the proportion of females with literacy was significantly correlated with the cholera incidence during the outbreak in Lusaka District, 2023-24. It might have been because the literacy was linked to the increased compliance to the guidance of the public health authorities, including those on the infection prevention measures [38]. Similar patterns were observed during the COVID-19 pandemic in Zambia in the early 2020, in which the increased incidence of the COVID-19 associated community deaths was associated with the proportion of individuals without education higher than the primary level in the area [24]. Our results might suggest the importance of enhanced risk communication and community engagement strategy for residents in those high-risk areas.

Limitation of our study include a limited proportion of cases with geocoordinate data. This was assumed to be due to the overwhelming number of cholera cases against the intensive case investigations, including the collection of geocoordinate data, conducted as part of the outbreak response by MOH and ZNPHI. However, the proportion of cases with and without geocoordinate data did not vary significantly by the month and subdistrict of patient identification. Therefore, it was assumed that the effects of the limited proportion of cases with geocoordinate data on the temporal and geographical composition of the dataset were minimal.

## Conclusions

Our study reported that the cholera incidence was significantly higher in unplanned residential areas. Based on the findings of this study, inadequate access to WASH facilities was assumed to be associated with such geographical distribution during the outbreak in Lusaka District, 2023-2024. Continuous efforts are warranted to improve the water and sanitation systems and public health intervention strategy for those high-risk areas.

## Supplementary Information

Supplementary material 1: Table 1. Definitions of environmental factors produced using the data from the demographic health survey (DHS) 2018. Table 2. Dataset of cholera-suspected cases in Lusaka District, 2023-24.

## Data Availability

Dataset used for this study will be shared by the study group with a reasonable request.

## Abbreviations

WAS: Water, sanitation, and hygiene
MOH: Ministry of Health
ZNPHI: Zambia National Public Health Institute
IDSR: Integrated Disease Surveillance and Response
ORP: Oral rehydration point
DMMU: Disaster Management and Mitigation Unit
EW: Epidemiological week
IQR: Interquartile range
RR: Relative risk
CATI: Case-area target intervention
COVID-19: Coronavirus disease 2019
