# Supplementary material for: Geospatial analysis of cholera outbreak in Lusaka, Zambia, between 2023 and 2024
Source: Trop Med Health. 2025 Mar 28;53:42. doi: 10.1186/s41182-025-00718-4 (PMC11954341; doi:10.1186/s41182-025-00718-4)
Supplement: Supplementary file 1 — Supplementary material 1: Table 1. Definitions of environmental factors produced using the data from the demographic health survey (DHS) 2018. Table 2. Dataset of cholera-suspected cases in Lusaka District, 2023-24. [file 41182_2025_718_MOESM1_ESM.docx]

**Supplementary data**

Supplementary table 1. Definitions of environmental factors produced using the data from the demographic health survey (DHS) 2018

| Environmental factors | Definitions |
| --- | --- |
| Population density [/10^3^ km^2^] | Number of residents (/1,000) divided by the area size (km^2^) |
| Proportion of females with literacy | Number of female residents who are able to read at least parts of sentence divided by total number of female residents in the area |
| Proportion of males with literacy | Number of male residents who are able to read at least parts of sentence divided by total number of male residents in the area |
| Proportion of individuals without soap/detergent at home | Number of residents who do not have any soap or detergent at home divided by total number of residents in the area |
| Proportion of individuals without piped-in drinking water at home | Number of residents who do not have piped-in drinking water at home divided by total number of residents in the area |
| Proportion of individuals without water for hand washing at home | Number of residents who do not have water for hand washing at home divided by total number of residents in the area |
| Proportion of individuals who require more than 30 minutes to obtain water | Number of residents who require more than 30 minutes to obtain water divided by total number of residents in the area |
| Proportion of individuals who share toilets with others or do not have toilets at home | Number of residents who share toilets with other houses or those who do not have toilets at home divided by total number of residents in the area |

Supplementary table 2. Dataset of cholera suspected cases in Lusaka District, 2023-24

|  | Patients total  (n=16,146) | Patients with geocoordinate data  (n=4,591) | Patients without geocoordinate data  (n=11,555) |
| --- | --- | --- | --- |
| Month of patient identification | | | |
| October, 2023 | 557 (2.2) | 83 (1.1) | 474 (2.7) |
| November, 2023 | 592 (2.4) | 189 (2.6) | 403 (2.3) |
| December, 2023 | 2,479 (15.4) | 901 (19.6) | 1,578 (13.7) |
| January, 2024 | 10,307 (63.8) | 2,783 (60.6) | 7,524 (65.1) |
| February, 2024 | 2,180 (13.5) | 626 (13.6) | 1,554 (13.4) |
| March, 2024 | 31 (0.2) | 9 (0.2) | 22 (0.2) |
| Sub-district | | | |
| Chawama | 1,067 (6.6) | 71 (1.6) | 996 (8.6) |
| Kabwata | 1,247 (7.7) | 367 (8.0) | 880 (7.6) |
| Kanyama | 4,969 (30.8) | 1,789 (39.0) | 3,180 (27.5) |
| Lusaka Central | 600 (3.7) | 118 (2.6) | 482 (4.2) |
| Mandevu | 992 (6.1) | 294 (6.4) | 698 (6.0) |
| Matero | 4,595 (28.5) | 1,134 (24.7) | 3,461 (30.0) |
| Munali | 2,676 (16.6) | 818 (17.8) | 1,858 (16.1) |

Number (%) were indicated.
